# Interactions between membrane-bound streptococcal alpha-enolase and human plasminogen captured through cryogenic-electron microscopy

**DOI:** 10.3389/fmolb.2025.1666748

**Published:** 2025-09-24

**Authors:** Sheiny Tjia-Fleck, Bradley M. Readnour, Zhong Liang, Yetunde A. Ayinuola, Francis J. Castellino

**Affiliations:** Department of Chemistry and Biochemistry and W. M. Keck Center for Transgene Research, University of Notre Dame, Notre Dame, IN, United States

**Keywords:** plasminogen binding proteins, cryogenic electron microscopy, *Streptococcus* pyogenes enolase, tissue-type plasminogen activator, scanning electron microscopy

## Abstract

Certain invasive strains of the Gram-positive bacterium *Streptococcus pyogenes* exploit human plasminogen (hPg) to promote tissue invasion and pathogenesis. hPg is a single-chain multi-modular zymogen containing five kringle domains (K1-K5), four of which interact with lysine or pseudo-lysine residues on binding partners, positioning hPg for activation to plasmin and enhancing bacterial dissemination. The major hPg binding protein in *S. pyogenes* is the multicopy surface-resident M-protein, or other surface proteins, such as the homooctameric glycolytic enzyme, enolase (SEn). SEn lacks features for direct translocation from the cytoplasm to the bacterial surface, and it is unclear how Sen is translocated to the bacterial surface. Additionally, the mechanism by which SEn binds hPg is poorly understood. In this study, we show that SEn is exported via lipid microvesicles (MV), likely originating from the cytosolic membrane. Using cryogenic-electron microscopy, we provide a high-resolution (<3.4 Å) map of SEn reconstituted into dioleoyl phosphatidylglycerol (DOPG) liposomes, which serves as our MV model. The Sen-DOPG map reveals that two subunits of the SEn octamer are exposed to the extracellular medium, while six remain inserted within the membrane or vesicle interior. However, this interaction does not induce a conformational change in hPg, which remains in a closed conformation, thereby limiting the SEn stimulatory effect on many hPg activators, except for host tissue-type plasminogen activator (tPA). Instead, the ability of SEn to bind tPA is the primary factor driving enhanced hPg activation. These findings highlight a novel mechanism by which MV-associated SEn promotes hPg activation preferentially through tPA, independent of a hPg conformational rearrangement.

## 1 Introduction

Gram-positive *Streptococcus pyogenes* (GAS) infections, which range from invasive to self-limiting, and their sequela, *e.g.,* rheumatic heart disease, represent a major cause of world-wide morbidity and mortality. There are >500,000 deaths per year globally attributed to invasive *S. pyogenes* diseases (Carapetis et al., 2005). In addition, noninvasive infections cause significant morbidity with societal and economic consequences that result in costs exceeding $500 M annually (Pfoh et al., 2008). There are no available universally effective vaccines for these infections.

Given the serious nature of *S. pyogenes* infections, many studies have focused on identifying the virulence determinants of this microbe. The surface of *S. pyogenes* is studded with numerous proteins that are stabilized on the surface through covalent and noncovalent interactions with the cytosolic membrane, peptidoglycan cell wall, and outer capsule. Most studies agree that the multicopy cell surface M-protein (M-Prt/PAM), translated from the *emm* gene, is an important factor in the severity of *S. pyogenes* infections. These *S. pyogenes* strains can be further placed into five classes (A-E), based in part on the number and organization of a short polycistronic group of five consecutive polycistronic genes within the single component *mga* regulon, viz.*,* [mga-fcR-enn-emm-scpA], transcribed by Mga, all of which requires a Sortase A (SrtA)-catalyzed post-translational cleavage for their proper translocation (Bessen et al., 1997). The studies presented herein focus on the highly prevalent and virulent Pattern D class which contains all of these genes (McGregor et al., 2004). Common among these Class D M-Prts is their invariant ability to directly bind host human plasminogen (hPg) at high affinity (low nanomolar). This is the only class of *S. pyogenes* in which the M-Prt directly interacts with hPg at biologically meaningful affinities. Some members of other *S. pyogenes* classes, *e.g.,* A-C, contain M-Prts that can directly interact with fibrinogen (Fg) and in-turn assemble hPg on their surfaces via Fg binding.

Also present on the surface of all *S. pyogenes* strains are the moonlighting glycolytic enzymes, glyceraldehyde-3-phosphate dehydrogenase (GAPDH) (Pancholi and Fischetti, 1992) and α-enolase (SEn) (Pancholi and Fischetti, 1998), the latter of which has been promoted as an important hPg binding protein on cell surfaces, at least in mammalian cells (Miles and Ellis, 2003). Enolase (EC:4.2.1.11), a highly conserved metalloenzyme functioning in glycolysis and gluconeogenesis, is present in nearly all cells and is constructed as a homooctamer of homodimers, having four major and minor interfaces in most prokaryotes, but exists as a homodimer in higher organisms. The manner in which SEn translocates to the *S. pyogenes* surface from the cytoplasm is unknown to now since SEn does not possess a signal sequence, a transmembrane sequence, a cell wall anchoring motif, or any other known translocation signal.

Mature hPg (Glu^1^-hPg) is a 791-residue single-chain modular zymogen of molecular weight ∼92,000. The mature protein consists of a NH_2_-terminal 77-residue activation peptide (AP), followed consecutively by five triply disulfide bonded kringle domains (K1-K5, respectively), each containing ∼80 amino acids, four of which bind to an important mediator, lysine. Located downstream of the kringle domains is a short activation loop, containing a peptide bond, Arg^561^-Val^562^, that is necessarily cleaved by all hPg activators. This step results in the formation of plasmin (hPm) (EC 3.4.21.7) which is composed of a heavy chain (residues 1–561), containing all five hPg kringle regions, that is doubly disulfide linked to a light chain (residues 562–791). This light chain is homologous to serine protease (SP), such as trypsin (Castellino, 1984), that confers upon hPm a strong tryptic-like proteolytic activity.

Except for K3-hPg, the kringle regions of hPg have variable abilities to interact with lysine and its analogues (Castellino, 1984). This property enables hPg and hPm to interact with several hPg/hPm surface binding proteins that contain COOH-terminal lysine residues, *e.g.,* enolase, and/or internal through-space lysine isosteres with appropriately rigidly placed i, i+3 NH_2_- and COOH-side chain residues of Lys and Asp/Glu, as is the case with Class D streptococcal M-proteins (Cnudde et al., 2006; Wang et al., 2010; Yuan et al., 2017). A number of hPg binding proteins on cells are constructed in this manner, providing cells with the ability to conscript hPg, catalyze activation of this zymogen, with *S. pyogenes*-secreted streptokinase (SK), and stabilize hPm on cells (Bhattacharya et al., 2012; Miles et al., 2005). Other than *S. pyogenes*-secreted SK, hPg can be activated using host plasminogen activators, *i.e.,* tissue-type plasminogen activator (tPA) and urokinase-type plasminogen activator (uPA). hPm can then be employed by cells to disseminate and invade the host *via* cell surface hPm (Didiasova et al., 2014).

**FIGURE 1:**
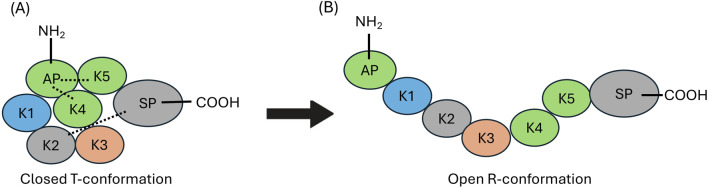
Structural organization of human plasminogen (hPg). **(A)** The closed (T) conformation of hPg illustrating the interactions between AP, with the K4, and K5 domains, as well the interaction between K2 and the SP domain those of K2 and the SP domain (dotted lines). **(B)** The open highly activatable relaxed (R) conformation of hPg highlighting the loss of intramolecular interactions that maintain the closed conformation.

These kringle lysine binding sites (LBS) also confer on hPg important intramolecular interactions, particularly those between K1, K4, and K5, and lysine isosteres in the AP, as well as interactions between K2 and the SP, that are responsible for maintaining a tight (T) conformation (Figure 1, left) of the poorly activatable zymogen. When this conformation is relaxed (R) (Figure 1, right) by natural proteolytic removal of the AP, generating (Lys^78^-hPg), or by competition with lysine analogs in solution (Brockway and Castellino, 1972; Violand et al., 1978) or other proteins (Zhang et al., 2003; Ayinuola et al., 2020; Ayinuola and Castellino, 2023), hPg becomes a highly activatable protein.

These considerations dictate that biologically relevant hPg/hPm binding proteins should be identified and structurally characterized in order to identify manners in which the binding of hPg to pathological cells can be disrupted. These biologically relevant binding partners often influence the conformation of hPg in order to stimulate its activation in the presence of plasminogen activators. While the importance of enolase has been studied in several cell types, this has not been the case in *S. pyogenes* cells, especially in strains where another important multicopy cell surface hPg binding M-protein (PAM), is present. In this communication, we have investigated the translocation of SEn to the cell surface and identified the manner of its interaction with hPg and its activation using tPA as a critical starting point for understanding the importance of SEn in *S. pyogenes* virulence.

## 2 Materials and methods

### 2.1 Protein expression and purification

The cDNAs encoding recombinant *sen* (GenBank: AMY97107.1) from *S. pyogenes* strain AP53 cDNA (Bao et al., 2016) was expressed in *Escherichia coli* and purified as described (Ayinuola et al., 2022).

Recombinant human tissue-type plasminogen activator (tPA) (EC 3.4.21.68), expressed in CHO cells, was obtained from Genentech (San Francisco, CA) and confirmed by assay to be suitable for experimental use.

### 2.2 Scanning electron microscopy (SEM) of *S. pyogenes-*AP53 cells

The complete procedure for scanning electron microscopy (SEM) imaging of *S. pyogenes*-AP53 cells was operationally adapted from our previous work (Readnour et al., 2022). In summary, 4 mL of overnight *S. pyogenes*-AP53 single cultures, grown in Todd-Hewitt Broth/1% yeast extract (THY), was inoculated into 40 mL of THY, which was then grown for ∼2.5 h to mid-log growth phase (OD_600 nm_ ∼0.55). The cells were then subjected to centrifugation at 6,000 rpm, after which the supernatant was removed and the remaining cells were washed 3x with phosphate-buffered saline, pH 7.4 (PBS). The cells were then fixed with 2% glutaraldehyde/0.1 M sodium cacodylate in PBS. Next, an aliquot of 2 µL was applied to glass microscope slides coated with 0.01% poly-L-lysine, and allowed to cross-link for 1 h. The slides were fixed for a second time by submersion in 1% OsO_4_ in PBS for 1 h. The sample slides were then dehydrated stepwise with increasing concentrations of ethanol (50%, 70%, 80%, 95%, 100%) for 10 min each, and then submerged in liquid CO_2_ and allowed to reach critical points to dry. Glass slides were then attached to 25 mm SEM stubs and gold sputter coated to a thickness of 3 nm. The samples were then imaged on a Magellan 400 SEM.

### 2.3 Isolation and mass spectrometry (MS) of *S. pyogenes* extracellular microvesicles (MV)

Overnight *S. pyogenes*-AP53 cultures (40 mL) were used to inoculate 400 mL THY, which was grown to mid-log phase (OD_600 nm_ ∼ 0.55). The cells were centrifuged for 10 min at 6,000 rpm. The culture supernate was filter-sterilized by passage through a 0.22 µM filter, followed by ultrafiltration of the filtrate through a 500 kDa MW cut-off membrane to remove soluble proteins. The *S. pyogenes* MVs retained on the membrane were suspended in 4 mL PBS, and the filtrate was concentrated to 4 mL by diafiltration through a 30 kDa MW cut-off Amicon spin filter. Both fractions were stored at 4 °C. An aliquot (100 μL) of the MVs was digested overnight with MS-grade trypsin gold (Sigma-Aldrich) at 37 °C and the reaction was terminated by 1% TFA. The samples were then passed over a C18 desalting column equilibrated with 5% acetonitrile/0.5% TFA, and finally eluted with 70% acetonitrile in H_2_O. The samples were applied to a Bruker Impact II UPLC system, and the fractions were processed by MS on a Bruker Impact II Very High-Resolution Quadrupole Time-of-Flight Mass Spectrometer. The fractions were analyzed by PEAKS software (Zhang et al., 2012) to determine the correlated *S. pyogenes*-AP53 protein by identifying its corresponding mass fractions, coverage, and to quantify peak area/abundance.

### 2.4 Integration of SEn into DOPG vesicles and flow cytometry analysis

Phospholipid vesicles were prepared as previously described (Tjia-Fleck et al., 2023) with the following changes. DOPG (1,2-dioleoyl-sn-glycero-3-phosphoglycerol sodium salt; Sigma-Aldrich) was dissolved in CHCl_3_ and then dried into a film using a rotary evaporator. The resulting dried DOPG film was resuspended to a final concentration of 0.1 mM in 10 mM sodium phosphate, pH 7.4, and sonicated for 3 min per 3 mL DOPG suspension. The vesicles were frozen at −20 °C for 3 days, thawed slowly at 4 °C overnight and sonicated the following morning. The overall freeze-thaw process was repeated four times, after which the vesicles were stored at −20 °C until further use.

To constitute SEn into DOPG vesicles, several 200 μL aliquots of thawed DOPG vesicles were first blocked with 2.5% BSA and incubated for 2 h at ambient temperature. The multilamellar vesicles were then pelleted by centrifugation at 16,000xg for 13 min (Temmerman and Nickel, 2009), resuspended in either 10 μM SEn/PBS (experimental) or in PBS alone (blank) and incubated for 1 h. After this step, the mixture was subjected to a 30 s sonication and the SEn concentration in the experimental tube was adjusted to 15 μM, while the blank was volume-matched with PBS. Following an additional 1 h incubation, the vesicles were pelleted as above, washed once with PBS, and incubated with polyclonal rabbit anti-SEn for 30 min. After one wash step, the vesicles were incubated with Alexa Fluor 488-chicken-anti-rabbit IgG (Invitrogen) in the dark for 30 min. Finally, the vesicles were washed, pelleted, and resuspended in 200 μL PBS. SEn-constituted DOPG vesicles were analyzed for fluorescence by flow cytometry. The data were acquired at a flow rate of 10 μL/min, with 10,000 events per run on BD FACAria III (BD Biosciences). DOPG vesicles were gated based on FITC-A fluorescence vs*.* side-scatter, both displayed on logarithmic scale. Dot plot histograms were analyzed using FCS Express version 7. FITC positive populations were defined relative to SEn blank controls, with DOPG vesicles considered positive if their fluorescence intensity exceeded that of the control.

### 2.5 Evaluation of hPg/tPA binding to SEn-constituted DOPG

For aliquots used to investigate the interaction of SEn-constituted DOPG vesicles with hPg or tPA, the incubation steps with polyclonal rabbit-anti SEn and Alexa Fluor 488-chicken-anti-rabbit IgG were omitted and the tubes were incubated with hPg (0 or 0.8 μM in PBS) or tPA (0 or 0.8 μM in PBS) for 1 h as previously described (Ayinuola et al., 2022). In this experiment, the blank included DOPG vesicles incubated without SEn, but incubated with 0.8 μM hPg or tPA, and DOPG vesicles incubated with SEn without hPg or tPA. The DOPG vesicles in the tubes were pelleted, washed once and incubated with monoclonal mouse-anti hPg (ERL, South Bend, IN) or mouse-anti tPA (Santa Cruz Biotechnology), and incubated for 30 min. After a single wash, the samples were incubated with Alexa Fluor 488-donkey-anti-mouse IgG (Invitrogen) in the dark for 30 min. Finally, each sample was pelleted, washed, and resuspended in 200 μL PBS. hPg or tPA bound to SEn-constituted DOPG vesicles was analyzed by flow cytometry as described above.

### 2.6 Activation of hPg by DOPG-SEn-tPA complex

SEn-constituted DOPG and SEn-blank DOPG vesicles were prepared as described above. Both samples were incubated with 0.8 μM tPA at ambient temperature for 1 h, followed by centrifugation at 16,000 × g for 13 min to pellet the vesicles. The samples were washed 3x with 1 mL PBS and centrifuged between washes to remove supernatant. After the final wash, pellets were then resuspended in 200 µL PBS. Aliquots of 5 μL and 10 μL were then used to accelerate reaction mixtures containing 200 nM hPg and 0.25 mM H-D-Val-l-Leu-l-Lys-p-nitroanilide dihydrochloride (S2251; Diapharma, West Chester, OH) in 100 mM HEPES/150 mM NaCl. The absorbance at 405 nm (Abs_405nm_) was continuously monitored for 1 h, and plots of Abs_405nm_ vs*.* t^2^ (min)^2^ were generated with GraphPad Prism 10.0. The initial rates of hPm formation were calculated as described earlier (Chibber et al., 1985).

### 2.7 Cryo-EM analyses of SEn-constituted DOPG with or without hPg

For cryo-EM analysis of SEn-constituted DOPG vesicles with or without hPg, the procedure described above was followed for sample preparation except that BSA blocking step and the incubations with primary and secondary antibodies were omitted. After the final wash step, samples were resuspended in 100 μL per 2 mL of original DOPG starting volume and extruded through a 0.05 μm Nuclepore track-etch membrane (Cytiva) to generate small unilamellar vesicles (SUV).

The extruded samples were quickly pipetted on the glow-discharged CF-1.2/1.3 Gold Ultrafoil® 300 mesh grids (Quantifoil, Jena, Germany). The grids were blotted for 12 s at blot force setting of 14 at 25 °C/100% humidity. A total of three grids were collected at different time points and pooled for data processing. The grids were vitrified by plunge-freezing in liquid ethane using the FEI Vitrobot MK IV (ThermoFisher, Waltham, MA) and loaded into the FEI Titan Krios transmission electron microscope (TEM). Data collections were performed at 300 kV using the Gatan (Pleasanton, CA) K1 and K3 direct electron detector with a Gatan Quantum GIF energy filter. The resulting images were processed using SerialEM (Boulder Labs, Boulder, CO) or EPU (ThermoFisher) software at a nominal magnification of ×81,000, resulting in a calibrated pixel size 1.078 Å and 1.068 Å for data collected from Gatan K1 and K3, respectively, for DOPG-Sen, and 0.539 Å for DOPG-Sen-hPg. The total exposure dose used was 53.7 e^–^/Å^2^ and 60 e^–^/Å^2^, respectively, with a spherical aberration coefficient of 2.7 mm.

All image processing was accomplished as previously described (Tjia-Fleck et al., 2023) with the following changes. Image processing was performed through Cryosparc v4.2.1 software (http://www.cryosparc.com). References for each data collection were obtained at every initial run. The images were dose-weighted and potential drift was removed using Patch Motion correction and patch Contrast Transfer Function (CTF) extraction, which was performed as previously described (Tjia-Fleck, et al., 2023). Curate exposure for each data set was performed to remove thick crystalline ice and any undesirable micrographs that would hinder particle picking.

An initial collection of 4,773 micrographs was curated down to 4,406 micrographs to obtain the cryo-EM map of DOPG-SEn-hPg. An initial set of ∼4,000,000 particles was selected, and a 2D classification of these particles resulted in 50 classes that were then curated down to 19 classes. This data set was then refined using template picker to reach a final particle count of 1,043,328 spread across 12 classes. An *ab initio* reconstruction and 3D refinement were performed using these particles to obtain the final map of hPg bound to SEn in DOPG vesicles.

### 2.8 Model fitting and refinement to the cryo-EM map

Initial modeling of hPg bound to DOPG-SEn was performed using AlphaFold P00747 (https://pubchem.ncbi.nlm.nih.gov/protein/P00747), as templates. Phenix 1.2.1 (https://phenix-online.org/) real space refinement was used to align the template model to the electron density of hPg in the cryo-EM map. For regions that were poorly aligned with the map, Phenix was used to generate these regions, and molecular replacement was used to fit these areas within the existing model. The model was further real space refined to best fit the experimentally determined map, and measured for rotamer and Ramachandran outliers using ChimeraX ISOLDE (https://tristanic.github.io/isolde/). Outliers were removed and replaced to maximize the number of preferred conformations and to best align with the cryo-EM experimental map.

Placement of SEn dimer in hPg fully bound to SEn in DOPG vesicles were done through visual placement using ChimeraX. The SEn major interface from the soluble SEn structure (PDB:7UGU) (blue and red, chain a and b) was placed in the dimer shaped electron density with a size that tapered from 66 to 77 Å, while the minor interface (red and green, chain b and c) was placed in the dimer shaped electron density with the size of 82–90 Å.

### 2.9 Validation and FSC confirmation of final map and model

Resolutions of cryo-EM maps for DOPG-SEn and DOPG-SEn-hPg were confirmed using the gold standard FSC (0.143) calculation (Supplementary Figure S4). Validation of hPg model was performed using the Phenix validation tool and further confirmed using EMDB and PDB. The maps and the hPg model were deposited in EMDB and PDB, respectively. EMDB entries include EMDB:42,435, EMDB:42,441, and EMDB:42,462 for the DOPG-SEn major interface, combined interface, and DOPG-SEn-hPg, respectively. The PDB identity for hPg is 8UQ6. Values for the full validation are contained in Supplementary Table S1.

### 2.10 Dot blots of tPA and hPg-binding to SEn

SEn (2 μg, 1 μg, and 0.5 μg) was blotted onto nitrocellulose membranes and dried for 15 min at RT. Each membrane was blocked using 5% milk in Tris-buffered saline with Tween 20 (TBST) buffer for 1 h, then incubated with 0 nM, 800 nM tPA, or 800 nM hPg, followed by tPA antibody or hPg antibody. Lastly, the membranes were incubated with the respective secondary antibody conjugated to horseradish peroxidase and developed with *TMB (3,3′, 5,5′tetramethylbenzidine dihydrochloride*).

### 2.11 Binding of SEn and tPA using surface plasmon resonance (SPR)

SEn was immobilized onto CM5 chip as previously described (Ayinuola et al., 2022). tPA in 10 mM Na-Hepes/150 mM NaCl/3 mM EDTA/0.05% polysorbate-20, pH7.4 (HBS-EP+) buffer was used as the analyte at concentrations ranging from 0 to 2000 nM. The binding kinetics were evaluated under conditions identical to those used for SEn and hPg interactions with a flow rate of 10 μL/min, 600 s association time, 300 s dissociation time, and regeneration with 10 mM glycine-HCl, pH 1.5. The binding constants of triplicate experiments were affinity fit with the Biacore software.

## 3 Results

### 3.1 *S. pyogenes* extracellular lipid microvesicles (MV) contain SEn

**FIGURE 2:**
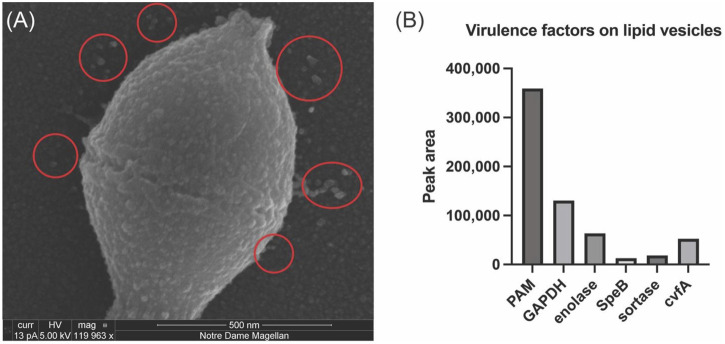
Analysis of *Streptococcus pyogenes*-AP53 (GAS)-derived MVs. SEM imaging and mass spectrometric analysis of isolated *S. pyogenes*-AP53 MVs. **(A)** SEM image of *S. pyogenes*, confirming the presence of MVs on the surface of *S. pyogenes*-AP53 cells (red circles). The large spherical structures on the surface of the cell and in the surrounding supernatant are representative of MVs commonly seen on the surface of *S. pyogenes*. **(B)** Mass spectrometric analysis of selected proteins found in isolated PL vesicles from of *S. pyogenes*-AP53. Five relevant GAS virulence factors are listed. These data represent independent experiments using separate MVs and do not differ by more than 15% for each protein.

*In vitro* experiments that incorporated SEn into DOPG vesicles produced a modestly higher stimulatory effect of SEn on hPg activation by tPA than SEn alone (Ayinuola et al., 2022; Tjia-Fleck et al., 2023). Since the cytoplasmic membrane of *S. pyogenes* contained ∼90% DOPG, and *S. pyogenes* cells have been shown to produce phospholipid vesicles which are integrated into the cell surface or released into the supernatant (Resch et al., 2016), the question arose as to whether SEn is present in the *S. pyogenes* extracellular MVs, which would perhaps explain the currently unknown mechanism of how SEn is transported from the cell cytoplasm to the cell surface. Herein, we showed by SEM, the presence of MV protrusions on the surface *S. pyogenes*-AP53 and in the surrounding growth medium of actively growing cells (Figure 2A). To confirm the presence of SEn on these MVs, mass spectrometric analysis was performed on proteolytic digests of the isolated MVs. The data showed that indeed SEn was present on the bacterial cell surface (Figure 2B), further suggesting that this is likely the mechanism by which SEn is transported to the *S. pyogenes* surface.

### 3.2 Cryo-EM map of DOPG-SEn

**FIGURE 3:**
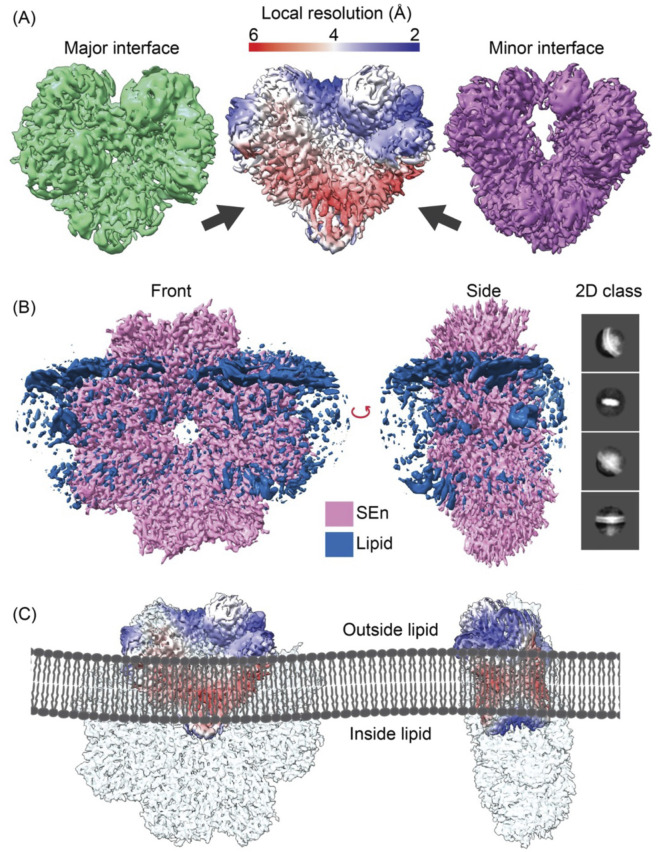
The DOPG/SEn dimer exposed on the DOPG surface. **(A)** The major interface (green), minor interface (purple), and the combined particle local resolution map (center), with the arrow pointing on the reddish region where PL interacts with the SEn map as shown in **(B)**. **(B)** Full cryo-EM map of full SEn with phospholipid obtained using a larger box size and C2 symmetry. The 2D class was obtained by extracting particles at 428 pix box size and circular mask diameter of 200 Å. **(C)** Models of PL/SEn dimers as they exist within the bilayers of the PL-vesicles. Left to right shows the octameric Sen with the major interface dimer exposed on the outside of the bilayer, the octamer of the exposed major interface dimer rotated 90°, and the octameric SEn with the minor interface dimer exposed on the outside of the bilayer.

In order to evaluate the manner in which SEn is oriented in DOPG vesicles, we performed cryo-EM analysis to visualize the structural organization of SEn within the vesicles (Supplementary Figure S1). Initial analysis of the cryo-EM particles obtained for DOPG-SEn showed two very distinct SEn orientations on the vesicle surface. In ∼88% of the particles, two subunits of the SEn octamers are exposed on the DOPG surface, while in the other ∼12%, six subunits are surface-exposed (Supplementary Figure S2). To generate the cryo-EM map of DOPG-SEn having two subunits exposed, a total of 4,599 curated micrographs were used for particle selection using a template picker obtained from the DOPG-SEn-hPg cryo-EM data, which was analyzed prior to that of DOPG-SEn. For the population with six subunits exposed, 1,191 curated micrographs were used. A total of 3,300,000 and 318,914 particles were obtained for DOPG-SEn, having two SEn subunits exposed, and six subunits exposed, respectively, of which the final 2D classification was performed. Out of the 50 classes obtained, 14 (dimer) and 7 (hexamer) classes with the best resolution were selected as 2D class templates. This resulted in a refined particle set consisting of 1,002,127 dimer particles and 73,781 hexamer particles as the final count. *Ab initio* reconstruction was performed separately for DOPG-SEn particles, having two SEn subunits exposed to solvent and six subunits exposed (Supplementary Figure S2). The best two initial volumes from heterogeneous refinement were used to further refine the data for DOPG-SEn having two subunits exposed, and the final map was obtained after further refinement with non-uniform refinement and local refinement, yielding a 3.4 Å map, shown in the center panel of Figure 3A. Further analysis of this final map showed that it is composed of a mixture of the major (Figure 3A-left panel) and minor (Figure 3A-right panel) interfacial dimers of SEn (Tjia-Fleck et al., 2023), where the major and minor refer to the surface buried between each subunit in an octameric SEn. Particles corresponding to each dimeric interface were re-classified and resolved to final resolutions of 3.2 Å and 3.9 Å, for major and minor interface dimers, respectively (Figure 3A-left and right panels). The final maps were then sharpened using the Phenix 1.20 Autosharpen map function as previously described. The local resolution of the focused SEn dimer map shows the region where the SEn dimer interacts with the DOPG particle (Figure 3A-red region center panel). Moreover, the full DOPG-SEn map clearly shows the region where the lipid interacts with SEn, as provided in Figure 3B. SEn predominantly exists on the vesicle surface at an angle such that two subunits are exposed outside of the vesicle, two subunits within the lipid bilayer and the other four subunits exposed into the lumen (Figure 3C).

### 3.3 Cryo-EM map and atomic modeling of hPg bound to SEn-constituted DOPG vesicles

We previously reported that hPg binding to DOPG-SEn is exclusively dependent on the presence of SEn, as DOPG vesicles lacking SEn did not generate detectable binding signals (Ayinuola et al., 2022). In the present study, we sought to investigate and clarify the locations of the amino acid residues that facilitate the SEn-hPg interaction. Hence, we solved the cryo-EM structure of hPg bound to SEn in DOPG vesicles.

**FIGURE 4:**
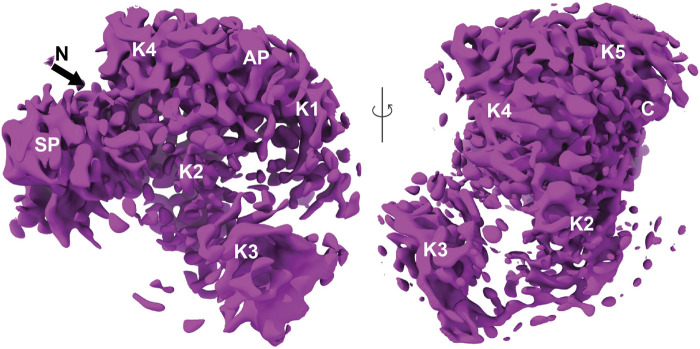
Finalized map and model of hPg bound to DOPG/SEn. The map of hPg bound to SEn on the surface of DOPG particles. Regions containing SEn were removed due to their lower resolution resulting from multiple binding orientations of hPg. The domains of hPg marked on the map show the characteristic kringle (K), activation peptide (AP), and serine protease (SP) domains.

There was a variation in the electron density of the SEn component of the DOPG-SEn-hPg, showing multiple potential binding sites for hPg, as denoted by the map generated, which shows multiple maps of two SEn subunits at different Kringle domains of hPg (Figure 4).

The hPg component assumes a more uniform orientation with well-defined electron densities. Thus, to characterize the hPg and separate these multiple binding sites, a 3D classification was performed and the class with the highest resolution was taken for further non-uniform and local refinement (Supplementary Figure S3A). Lastly, the map of hPg was finalized using the Phenix density modification function and was solved to a resolution of 3.0 Å (Supplementary Figure S3B) with the density map for two SEn subunits surrounding the masked hPg.

**FIGURE 5:**
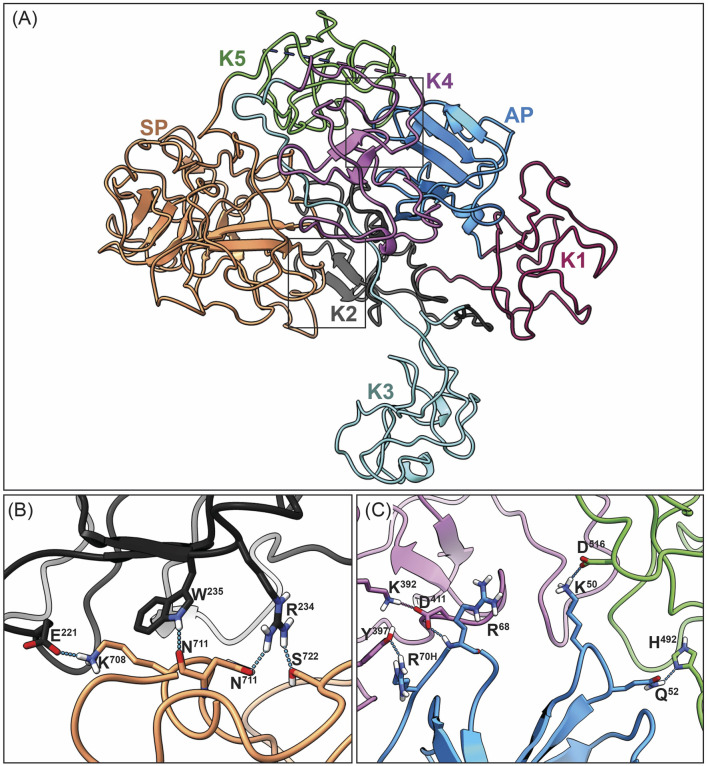
Overall closed conformation model of hPg bound to DOPG/SEn. **(A)** Overview of the hPg component of the DOPG/SEn/hPg complex with each Kringle domain colored separately: AP (blue), K1 (maroon), K2 (dark gray), K3 (cyan), K4 (purple), K5 (green), and SP (orange). The closed conformation of hPg is shown by the LBS interaction of **(B)** K2 and SP, and **(C)** K4, K5, with AP residues within the interior of hPg. In the cryo-EM hPg structure bound to SEn, these residues are close enough to interact with distance <3 Å, suggesting that hPg remains mainly in the closed conformation when bound to SEn.

**FIGURE 6:**
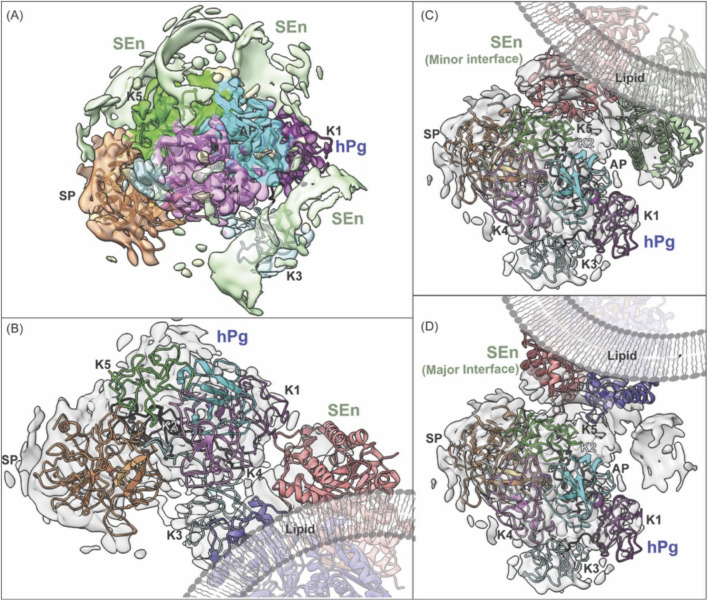
Fitting of SEn into cryo-EM density maps for the hPg-SEn complex in DOPG vesicles. **(A)** Overview of the full cryo-EM map showing hPg model fitted into its electron density with surrounding segmented densities attributed to SEn subunits shown in light green. **(B)** Masked hPg map focusing on the interactions of hPg K1 and K4 with SEn major interface. **(C)** Masked hPg map focusing on the interaction of hPg K5 and SEn minor interface. **(D)** Masked hPg map focusing on the interaction of hPg K5 and SEn major interface. Both types of interfaces spacing was found on the combined map, suggesting that different orientation of two subunits of SEn are capable of binding to hPg K5 domain. A lipid visualization has been added to enhance understanding.

The model design of hPg bound to DOPG-SEn was performed using a combination of Phenix and ChimeraX. An initial template model was taken using the AlphaFold algorithm (AF-P00747). Phenix real space refinement was used to maximize interactions between the model and the experimentally determined map. Phenix map-to-model was performed to fit regions within the AlphaFold model that could not be fitted to the map. These regions within the AlphaFold model were replaced and refitted to the map using real space refinement. Final refinement of DOPG-SEn bound hPg was optimized using the ChimeraX ISOLDE tugging function and the Trace and Build function. The final model of hPg bound to DOPG-SEn is shown in Figure 5A. Importantly, while the cryo-EM map for DOPG-SEn-hPg complex (EMDB:42,462) includes density corresponding to both hPg and SEn, only hPg was modeled and deposited in the corresponding atomic structure (PDB:8UQ6). Within the cryo-EM map of DOPG-SEn-hPg, multiple potential orientations were observed that showed electron density shapes that matched SEn (Figure 6A). This indicated that there are multiple possible binding orientations for SEn and hPg. The cryo-EM structure of soluble SEn (PDB: 7UGU) was placed into the three most prominent orientations to determine the potential interactions with SEn (Figures 6B–D).

### 3.4 Conformation of hPg when bound to SEn integrated in DOPG vesicles

The current cryo-EM model of hPg represents the 3D structure of hPg bound to SEn on the surface of a DOPG small unilamellar vesicle. The domain organization of hPg is depicted in Figure 1. Optimal hPg activation to hPm is often achieved following the relaxation of the closed hPg structure by elimination of intramolecular interactions that exist between the LBS housed in the kringle domains of hPg and side chains of its AP and SP domains (Ayinuola and Castellino, 2023). The elimination of these critical interactions by competitive ligands, such as the lysine analog, ε-amino caproic acid (EACA), generates large conformational changes in hPg that expose its activation loop for rapid conversion to hPm. In contrast, our cryo-EM structure reveals that despite being bound to SEn, hPg still retains the stabilizing intramolecular interactions between LBS, AP and SP domains (Figures 5B,C). Specifically, the K2 LBS residues D^221^, W^235^, R^234^, maintains contact with SP domain residues K^708^, the backbone oxygen of N^711^, and D^676^ respectively. Additionally, interactions between the NH_2_-terminal AP domain and the K4 and K5 kringle domains are preserved, including Q^50^ and D^516^, Q^52^ and H^492^, R^68^ and Y^397^, R^70H^ and D^411^/K^392^. These hydrogen bonds, all within <3 Å are consistent with those observed in the published X-ray structure of hPg in its closed conformation (Law et al., 2012). Together, these findings indicate that the binding of hPg to SEn does not induce a transition to the open (R) conformation.

### 3.5 Interactions between tPA and SEn

The observation that SEn fails to stimulate hPg activation reactions catalyzed by SK (Ayinuola et al., 2022) and uPA (unpublished data), but enhances those catalyzed by tPA (Ayinuola et al., 2022), raises the question of whether SEn serves as a binding surface for tPA. Notably, hPg activation was stimulated up to 8X higher when SEn was reconstituted in DOPG vesicles (Ayinuola et al., 2022). The resulting closed T-conformation of hPg further prompted us to re-examine the interactions between SEn and tPA. To clarify this, we probed the interaction of tPA with SEn and DOPG-SEn.

**FIGURE 7:**
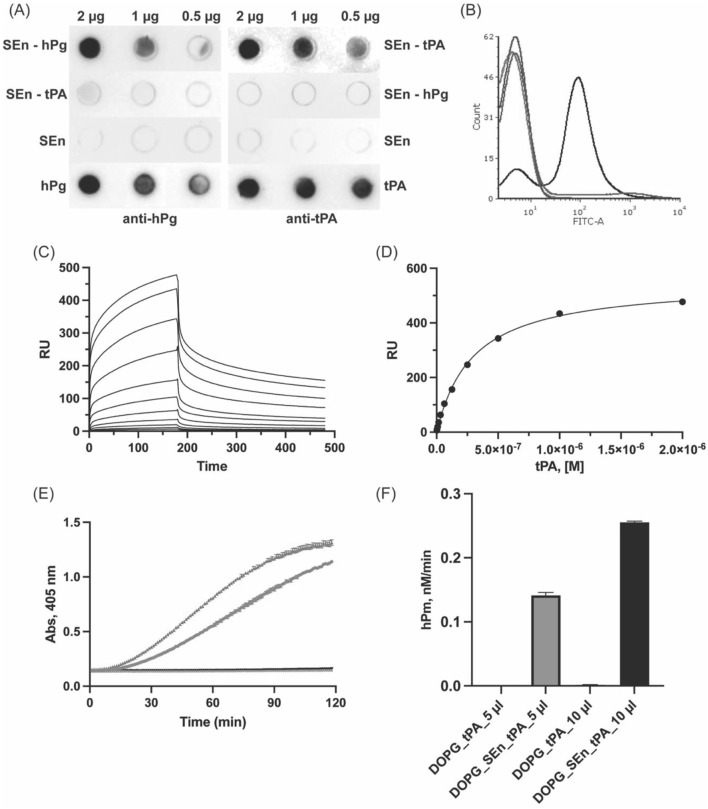
Interaction of tPA with SEn. **(A)** Dot blots of SEn (2 μg, 1 μg, 0.5 μg) incubated with either (800 nM) hPg or tPA and further incubated with anti-hPg or anti tPA. SEn, hPg, or tPA (2, 1, 0.5 µg) was blotted as a negative or positive control. **(B)** FACS analysis of tPA interaction with DOPG-SEn-tPA (black), showing a 70% ± 10% shift in the FITC axis, DOPG-SEn (red) is at <1%, DOPG-tPA (blue) is at 7% ± 1%, and DOPG alone (purple) is at <1%. **(C)** Representative SPR sensorgram for the binding of tPA to SEn. **(D)** Representative affinity fit between SEn and tPA shown with an average K_D_ of 161 ± 70 nM from six-plicate experiments. **(E)** Plots of Abs_405nm_ *versus* time, showing the effects of DOPG-SEn/tPA (5 μL-blue and 10 μL-green) and DOPG-tPA (5 μL-gray and 10 μL-black) on hPg activation. **(F)** Bar representation of the hPm generated by DOPG-SEn/tPA and DOPG-tPA.

Dot blot experiments of immobilized SEn or tPA, as a positive control, on nitrocellulose membrane show the ability of immobilized SEn to bind tPA in solution (Figure 7A). Negative controls show that SEn or SEn-hPg does not cross-react with tPA antibody, but a very faint cross-reaction was shown between SEn-tPA and hPg antibody, possibly due to Kringle 1 similarities. Moreover, FACS analysis shows that DOPG-SEn binds tPA, forming 70% ± 10% DOPG-SEn-tPA complex (Figure 7B). A quantitative analysis of the SEn-tPA interaction using SPR yielded a K_D_ ∼161 ± 70 nM. Notably, the sensorgram exhibited a biphasic dissociation phase (Figure 7C), which precluded a good fit using conventional kinetic models provided by BiAcore. Instead, the affinity model was employed to estimate the K_D_ by fitting the data to a C_50_ model (Figure 7D). To further demonstrate the interaction between SEn and tPA, we prepared DOPG-SEn-tPA by incubating DOPG-SEn with tPA, followed by extensive washing to remove unbound tPA. When used as an activator, the washed DOPG-SEn-tPA preparation effectively catalyzed hPg activation (Figures 7E,F). In contrast, DOPG vesicles lacking SEn but incubated similarly with tPA (DOPG-tPA) exhibited no detectable activity (Figures 7E,F).

## 4 Discussion

In addition to its well-known function as a central enzyme in the penultimate step in glycolysis in the cytoplasm, enolase has been found to participate in other processes in the cytoplasm, such as functioning as a heat shock protein in many types of cells (Iida and Yahara, 1985; Wilkins et al., 2002). Enolase is also located on the cell surface, where it interacts with hPg/hPm and displays hPm on cells (Pancholi and Fischetti, 1998), which can serve in normal functions of hPm, *e.g.,* fibrin surveillance, and in pathological processes, such as disruption of the extracellular matrix to aid in cellular invasion (Lu et al., 2011). Enolase can also function in the extracellular space microenvironment, where it is thought to be relevant in cancer phenotypes (Chung et al., 2023). The mode of transport to the cell surface of cytoplasmic glycolytic moonlighting proteins*,* such as enolase and GAPDH, among others, that do not contain signal sequences or other known translocation signals, has not been fully uncovered. This leads to a question as to the mechanism that allows proteins, such as enolase, to be found in other locations within the cell but also to be present in the extracellular space.

One of our interests in these systems involves the mechanism of transport of enolase to the surface of *S. pyogenes* cells, where it functions to recruit hPg/hPm and stabilizes the potent protease, hPm, on the surface of the bacteria. This proteolytic presence allows the bacteria to multiply and disseminate. To understand the significance of SEn in the recruitment of hPg to the *S. pyogenes* surface, especially in *S. pyogenes* strains that also contain more abundant and tighter hPg/hPm-binding surface proteins, we investigated the mechanism by which SEn is displayed on *S. pyogenes* cells and the functional properties of the bound hPg, especially its ability to become activated to hPm.

### 4.1 Role of lipid microvesicles (MVs) in the translocation of SEn

It has been previously reported that *S. pyogenes* cells produce MVs where the composition is rich in phosphatidylglycerols (Resch et al., 2016). Here, we showed by SEM imaging that, consistent with this previous report, *S. pyogenes*-AP53 produces distinct surface-attached lipid microvesicles (MVs), a portion of which is released into the extracellular environment of the bacteria (Figure 2A). Mass spectrometric analysis of MVs isolated from *S. pyogenes*-AP53 culture medium revealed that SEn was present in the released MVs (Figure 2B). This finding aligns with our earlier finding that a genetically modified *S. pyogenes* lacking PAM (plasminogen binding M-protein) exhibited markedly increased release of MVs and lacked surface-exposed SEn (26), reinforcing the idea that the primary mechanism by which SEn is retained on the *S. pyogenes* surface is through its association with MVs. Thus, we believe that SEn is displayed on the *S. pyogenes* surface through its incorporation into lipid vesicles that bud from the cytosolic membrane. The majority of these vesicles remain surface-attached in wild-type *S. pyogenes*-AP53, while a subset is released into the extracellular environment as free MVs.

In the genetically modified *S. pyogenes* strain, the correlation between the absence of surface PAM, the release of MVs, and the lack of surface-displayed SEn, suggests that PAM may contribute to MV tethering and thereby influence SEn localization. In this regard, an alternative mechanism is that microvesicular budding occurs at specialized membrane microdomains such as ExPortal, which is enriched in protein export and anchoring machineries that facilitate secretion and surface anchorage of proteins like PAM. This could explain the high level of PAM in the *S. pyogenes* AP53-derived MVs.

Overall, these findings indicate that MVs serve as key vehicles for SEn translocation, providing a novel mechanism for its extracellular presentation despite lacking traditional secretion signals. These insights prompted us to further investigate the nature of the interaction between SEn and phospholipid vesicles.

### 4.2 Orientation of SEn in DOPG phospholipid vesicles

Considering the hypothesis that MVs act as vehicles for the translocation of SEn from the cytoplasm, cryo-EM imaging was used to determine how SEn associates with MVs. Our strategy was to incorporate SEn into small vesicles of DOPG, which are close in size to MVs, and as such, appropriately model SEn in phosphatidyl-glycerol-rich natural MVs.

Cryo-EM reconstruction revealed the presence of the lipid density, which appeared as a diffuse outline, precluding definitive modeling of individual lipid molecules or precise protein-lipid interactions. Consequently, we were unable to determine the exact molecular contacts between SEn and the DOPG surface. However, based on the region of membrane-associated density and the orientation of SEn relative to the bilayer, our results show that SEn remains an octamer embedded in the DOPG bilayer, with two SEn protomers exposed outside. The other six protomers are embedded in the intravesicular space enclosed by the membrane (Figure 3). In a smaller percentage of the DOPG vesicles (∼12%), the opposite case was found, with SEn oriented in such a way that 6 subunits are exposed outside the vesicle (Supplementary Figure S2). Although SEn lacks a transmembrane domain, it exists on the phospholipid membrane, slanted upright. Surface charge mapping of the SEn octamer (Supplementary Figure S5) highlights regions of hydrophobic (gold) and hydrophilic (green) residues. The lipid density in DOPG-SEn (white mesh) is localized predominantly around hydrophobic surface patches, suggesting that membrane association is driven by hydrophobic contacts. However, hydrophilic residues are also abundant in this region, supporting a model in which a combination of localized hydrophobic clustering with the lipid tails, along with stabilization by hydrophilic interactions of positively charged residues with negatively charged phospholipid headgroups, anchors SEn on the MVs.

It was previously reported using atomic force microscopy that SEn is arranged parallel and flat on the DOPG membrane surface and binds to hPg with a stoichiometry of one hPg per SEn octamer (Balhara et al., 2014). Although it is possible for SEn to bind to hPg in this orientation, the previous study limits the orientation of SEn on the DOPG membrane due to the design of the experiment. More specifically, the vesicles seem to be disrupted, and the DOPG bilayer was deposited on an artificial (mica) surface, thus eliminating intravesicular solvent space for the SEn to orient properly on the membrane. We thus believe that the flat orientation proposed is artefactual.

### 4.3 Conformation of hPg in the SEn-hPg complex

A key regulatory mechanism of hPg activation involves conformational transitions between its closed and open forms, enhancing the generation of active plasmin. In solution, hPg predominantly adopts a closed, tight (T) conformation, but rapid activation requires a shift toward the relaxed (R) state, which exposes the activation loop and facilitates its conversion to plasmin (Violand et al., 1978). The crystal structure of the T-conformation reveals that this compact state is stabilized by intramolecular interactions involving several key residues within K2 and serine protease (SP) domains, as well as between K4, K5, and the activation peptide (AP) domains (Law et al., 2012). Notable interactions include D^219^ of K2-hPg and K^708^ of the SP domain; K^50^ of the AP and D^518^ of K5-hPg; and R^68^/R^70^ of the AP and D^411^/D^413^ of K4-hPg. While there is currently no structure for the R-conformation, numerous biochemical and biophysical studies have shown that ligand binding (Markus et al., 1979; Christensen and Molgaard, 1992; Marshall et al., 1994) and protein-protein interactions (Figuera-Losada, et al., 2009; Bhattacharya et al., 2014; Quek et al., 2023; Tang, et al., 2024) disrupt these stabilizing interactions, promoting the transition from the tight (T) to the relaxed (R) state with attendant rapid activation of hPg to hPm. In such cases, the activity of the plasminogen activator is said to be enhanced due to increased accessibility of the activation loop. The current cryo-EM structure of hPg bound to SEn shows that the critical intramolecular hydrogen bonds remain intact, indicating that hPg retains its T-conformation when bound to SEn immobilized on DOPG (Figure 5). This observation likely explains why SEn-bound hPg activation is not stimulated by activators like uPA and SK. However, hPg activation is stimulated by tPA possibly via mechanisms discussed below.

To further validate this structural observation, we compared our cryo-EM structure to the crystal structure of hPg (PDB:4DUR) (Law et al., 2012). Structural alignment revealed a high degree of similarity (RMSD = 10.57) in both secondary and tertiary structure. While the crystal structure is slightly more compact, likely due to packing effects and the complex composition of the mother liquid from which hPg was crystallized, the overall fold remains consistent, and key interactions that define the T-state are preserved.

These findings confirm that although hPg binds SEn immobilized on DOPG vesicles, it does not attain a relaxed R-conformation or expose its activation loop, which would greatly facilitate its activation. Thus, stimulation of hPg activation is restricted when hPg is bound to SEn.

### 4.4 SEn binding to tPA

Unlike uPA and SK, tPA is a relatively inefficient hPg activator in solution. However, upon binding to fibrin clots, the catalytic efficiency of tPA increase by nearly two orders of magnitude, primarily due to the colocalization of tPA and hPg on fibrin surface and a conformational shift in hPg that exposes its activation loop for cleavage (Collen and Lijnen, 2009). We observed strong binding between SEn and tPA (Figure 7), suggesting that a similar interaction may occur in this system. The high density of surface-exposed lysine residues on SEn may mimic fibrin, acting as a pseudo-clot that promotes tPA binding. Moreover, colocalization of hPg and tPA on SEn will impose a proximity effect that increases the local concentrations of the interacting molecules. These interactions likely contribute to the enhanced efficiency of hPg activation by tPA in the presence of SEn. However, the lack of similar enhancement in hPg activation by SK and uPA in the presence of DOPG-SEn (Ayinuola et al., 2022) suggests that passive colocalization alone is not sufficient for the stimulatory effect of SEn on tPA-catalyzed reactions and that tPA interaction likely plays a distinct mechanistic role.

### 4.5 Multiple binding sites between SEn and hPg

The experimental data obtained in this study show that the interaction between SEn and hPg is complex, likely due to the presence of multiple surface-exposed lysine residues on SEn that mimic C-terminal lysines capable of binding the kringle domains in hPg. Within the DOPG-SEn-hPg map (Figure 6), there appear to be multiple binding loci for hPg on SEn, as the electron density of SEn within the map shows that SEn adopts several orientations. Also, we observed that hPg can bind SEn through K1, K4, or K5. However, based on particle distribution and electron density, K1 seems to be the most preferred binding site. These multiple orientations serve as a limiting factor in our attempt to obtain a high-resolution structure of the SEn-hPg interface, thereby hindering the identification of the residues involved in the interaction on either protein. Our findings are consistent with previous molecular docking studies (Cork et al., 2015) that show an interaction between K1-hPg and residues located at the SEn minor interface dimers, including an interaction between K5-hPg and regions close to the previously predicted internal SEn residues K^252^ and K^255^ for hPg binding.

In this current study, although the cryo-EM map shows more than one hPg kringle domain capable of binding SEn, it is unlikely that K1, K4, and K5 bind simultaneously to SEn in a manner that relaxes hPg conformation, since hPg remains in a closed conformation. The observation that K1 is the predominant binding site, yet hPg does not transition to the open (R) state, supports earlier findings that K1 does not participate in changes that regulate hPg conformation (Ayinuola and Castellino, 2023).

## 5 Conclusion

In this work, we observed that, upon reconstitution in DOPG vesicles, two of the eight subunits of the octameric SEn are exposed. Both major and minor dimeric interfaces are equally distributed on the DOPG surface. Thus, hPg does not encounter the full octamer of SEn when added to the DOPG-SEn vesicle since SEn is mostly embedded in the membrane and interior space of the unilamellar vesicle. While we did not solve a model for SEn in complex with DOPG, it is plausible that conformational rearrangements contribute to its membrane association, particularly given that only surface-bound SEn efficiently binds hPg. Membrane attachment may promote subtle structural alterations that unmask binding sites for hPg, possibly enhancing the exposure of the C-terminal lysine critical for hPg binding.

Finally, we conclude that the transport of SEn to other locations in the cell is a result of its incorporation into MVs from the cytosolic membrane, which can then transport SEn to other locations in and out of the cell. Gram-positive bacteria have no outer cell membrane that would stabilize cell surface SEn and in these bacteria, we suggest that MVs from the cytosolic membrane transport, retain, and stabilize SEn on the surface. Thus, the manner in which SEn exists on a MV is a critical feature that requires a high level of understanding in order to propose its cell surface function in this important class of bacteria. This communication has contributed heavily to this deeper understanding.

## 6 Authors’ perspectives

It is very unlikely that functional hPg exists free in plasma. First, free hPg would be in a closed (T) conformation in solution that renders it highly refractive to activation, and second, free hPm, if formed in plasma, would be immediately inactivated by circulating inhibitors, such as α_2_-antiplasmin. Given the numerous hPg binding proteins in plasma and on cells and the cellular diversity of plasma, it is most likely that hPg and hPm function on cells, where it is known to be offered some protection against circulating inhibitors. The circulating activator of hPg, uPA, also has binding partners on cells, viz.*,* urokinase-type plasminogen activator receptor, thus optimizing the activation of hPg on cells by a soluble activator when hPg is bound to an appropriate partner. For hPg binding proteins to stimulate the activation of hPg, they must not only bind tightly to hPg and hPm, but in doing so, they must relax the T-conformation of hPg for optimal activation.

In *S. pyogenes* cells, there are a number of hPg binding proteins, but some have critical properties that are consistent with the requirement for establishing a proteolytic surface that is functional. Virulent strains of *S. pyogenes* contain surface M-proteins that can either bind hPg/hPm directly and tightly (∼1 nM), relax the conformation of hPg, stimulate its activation, especially by *S. pyogenes*-secreted streptokinase, and attenuate the propensity for inactivation of hPg by natural host inhibitors. Other virulent strains of *S. pyogenes* contain M-proteins that tightly bind fibrinogen, which in-turn interact tightly with hPg and stimulate its activation. These *S. pyogenes* strains are the most virulent in the *S. pyogenes* hierarchy. In these types of *S. pyogenes* cells, SEn or GAPDH do not fulfill these requirements and are unlikely to be important hPg-binding proteins. However, surface-bound SEn is perhaps important for other functions on cell surfaces and in MVs, which can carry cytoplasmic proteins to other regions of cells and to other cells. With our cryo-EM map revealing the structural organization of SEn in MVs, new paradigms are opened for investigators in many fields to dissect these issues.

Finally, our findings support a model where cytosolic SEn is incorporated into a blebbing membrane, which targets it to the *S. pyogenes* surface. Here, SEn can interact with binding partners like hPg. Moreover, the blebbing membrane is released as MVs containing *S. pyogenes* host protein binding factors, such as SEn. In MVs, as modeled by DOPG/SEn, SEn predominantly adopts an orientation that exposes two protomers of the octamer that bind hPg. Thus, we conclude that the transport of SEn out of the cytoplasm results from its incorporation into MVs from the cytosolic membrane which can then transport SEn to other locations inside and outside of the cell. Gram-positive bacteria have no outer cell membrane that would stabilize cell surface SEn, and in these bacteria, we suggest that MVs from the cytosolic membrane transport, retain, and stabilize SEn on the surface. Thus, the manner in which SEn exists on a MV, where it has the ability to function as a hPg binding protein, modulate cell adhesion, and participate in other processes, such as tissue remodeling, is a critical feature that requires a high level of understanding in order to propose its cell surface function in this important class of bacteria.

## Data Availability

The datasets presented in this study can be found in online repositories. The names of the repository/repositories and accession number(s) can be found in the article/Supplementary Material.
