# Supplementary material for: Interactions between membrane-bound streptococcal alpha-enolase and human plasminogen captured through cryogenic-electron microscopy
Source: Front Mol Biosci. 2025 Sep 24;12:1666748. doi: 10.3389/fmolb.2025.1666748 (PMC12504095; doi:10.3389/fmolb.2025.1666748)
Supplement: Supplementary file 1 [file DataSheet1.pdf]

Supplemental Information

Interactions between membrane-bound streptococcal alpha-enolase and human plasminogen captured through cryogenic-electron microscopy

Sheiny Tjia-Fleck, Bradley M. Readnour, Zhong Liang, Yetunde A. Ayinuola, and Francis J. Castellino

Supplemental Table S1. Validation and cryo-EM conditions for DOPG-SEn/hPg maps and hPg models, EMDB:42462, 42435, and 42441. Validation was performed using the Phenix validation tool and confirmed using official EMDB and PDB validation.

	**EMDB-42462**	**EMDB-42435**	**EMDB-42441**
Refinement Program	Cryosparc	Cryosparc	Cryosparc
Magnification	81,000	81,000	81,000
Voltage, kV	300	300	300
Electron Exposure, e-/Å^2	61.37	60	53.7
Defocus Range	-1.1 to -3.2	-1.2 to -2.9	-1.2 to -2.9
Pixel Size, Å	0.539	1.068	1.078
Initial particle images	1,748,783	3,300,000	3,300,000
Final particle images	1,042,328	1,002,127	1,002,127
Symmetry Imposed	C1	C2	C2
Resolution unmasked, FSC threshold 0.143Å	3.5	4.32	3.98
Resolution masked, FSC threshold 0.143Å	3	3.8	3.4
FSC threshold	0.143	0.143	0.143
Model resolution, Å	3.3	-	-
FSC threshold	0.5	0.5	0.5
Model resolution range	5.7	6	19.3
Map sharpening β factor (Å^2)	3.8	3.3	3.4
Refinement Program	Phenix, Chimera X	Phenix	Phenix
Number of atoms, non-H	6,930	-	-
Protein Residues	791	-	-
Ligands	0	-	-
Protein	31	-	-
Bond Length (Å)	0.006	-	-
Bond Angle (°)	1.066	-	-
MolProbity Score	2.55	-	-
Clash Score	18.83	-	-
Poor rotamers, %	0.15	-	-
Ramachandran favored, %	76.53	-	-
Ramachandran allowed, %	23.13	-	-
Ramachandran disallowed, %	0.53	-	-
Q-score	0.33	-	-

EMringer score	0.76	-	-

Supplemental Figure S1

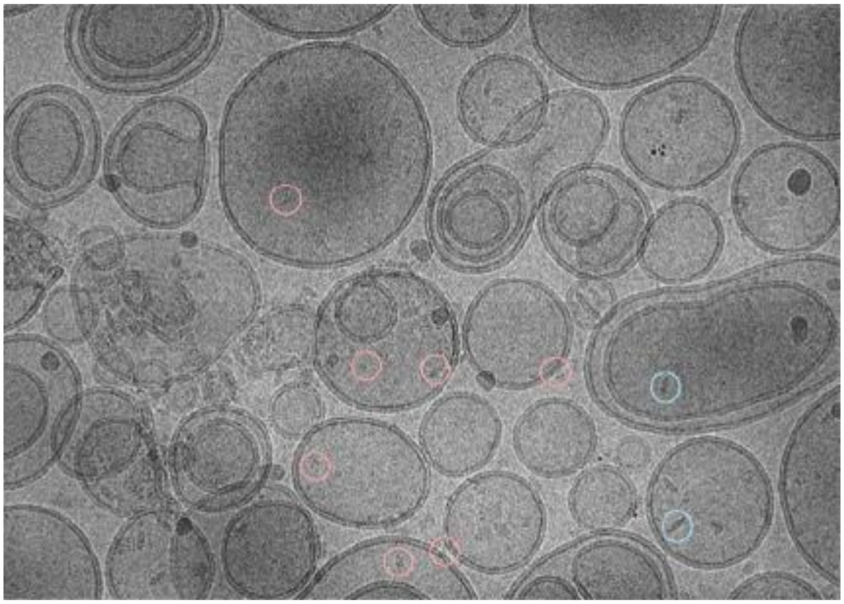

Supplemental Figure S1. PL-Sen-hPg micrograph for single particle analysis. Light red circles as representatives of two SEn subunits exposed on the surface and light blue circles when hexamer is exposed on the lipid surface.

Supplemental Figure S2

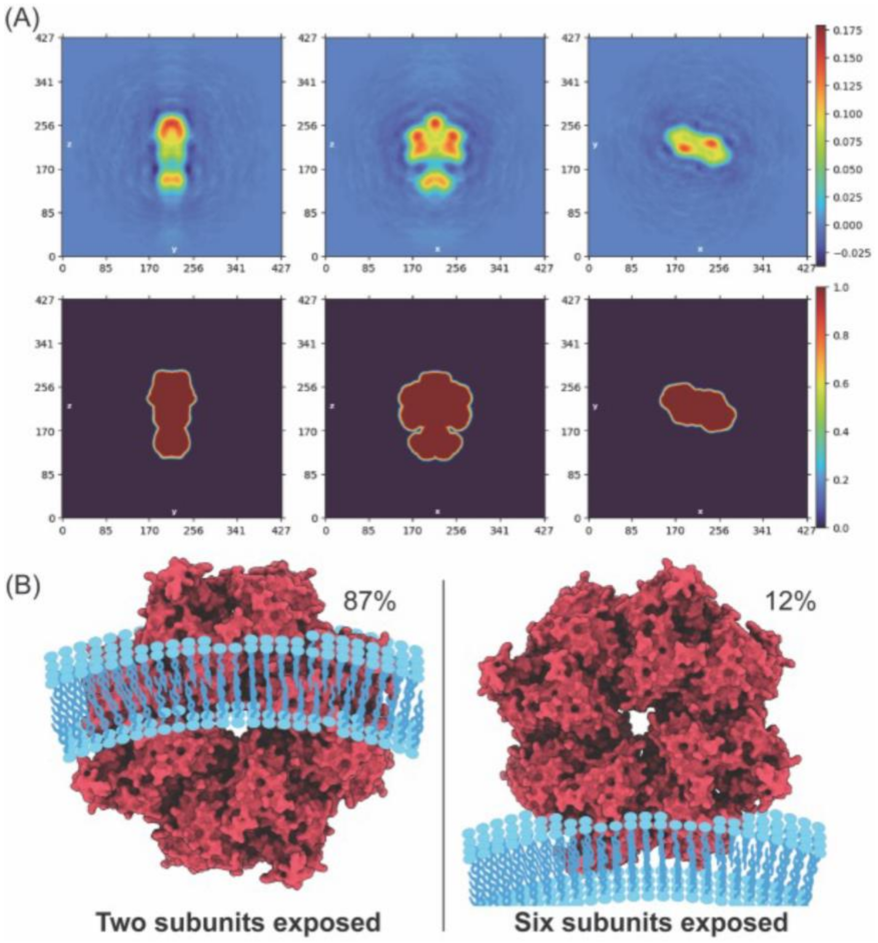

Supplemental Figure S2. (A). A small percentage of DOPG/SEn orientation (~12% total population) was found where the dimer was exposed inside the PL bilayer with hexamers exposed outside of the PL bilayer. Due to the small population size, the map of lipid SEn hexamers was not generated at high resolution. (B). Distribution of the SEn as observed, 87% of the population where two subunits of SEn are exposed outside of the lipid, and 12% of the population where six subunits of SEn are exposed outside of the lipid in cartoon rendition.

Supplemental Figure S3

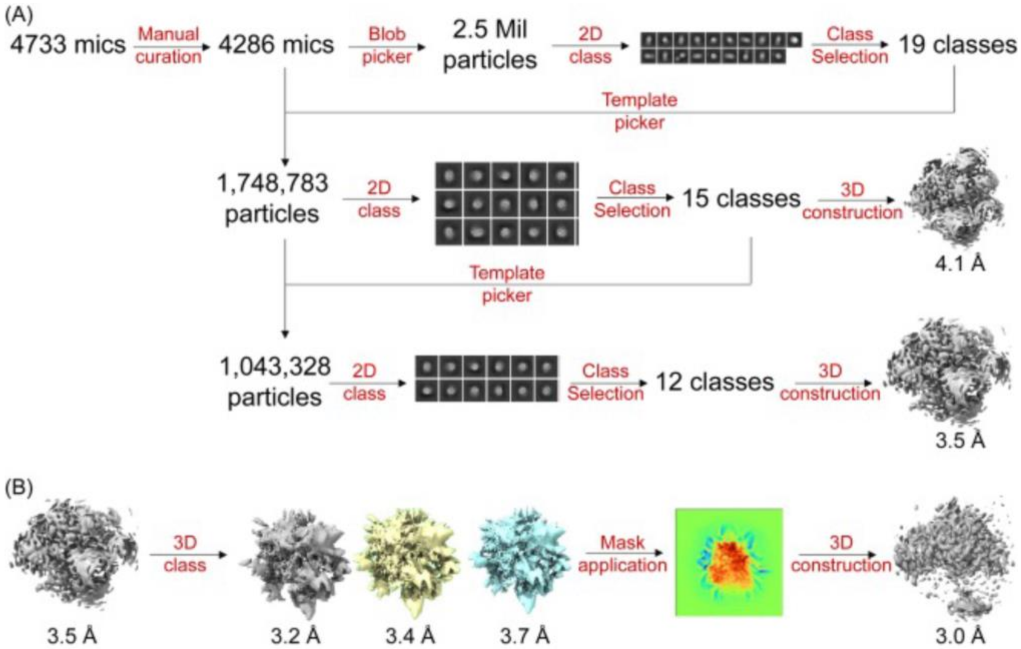

Supplemental Figure S3. **(A)** Flow chart detailing the process of obtaining the finalized DOPG-SEn-hPg map using Cryosparc with a deposited map EMDB-42462 at 3.5 Å. **(B)** The DOPG-SEn-hPg map was further refined by masking SEn electron density to obtain a 3.0 Å hPg map, which was used to fit the hPg model. A similar process was performed to obtain the maps of the DOPG-SEn exposed dimers.

Supplemental Figure S4

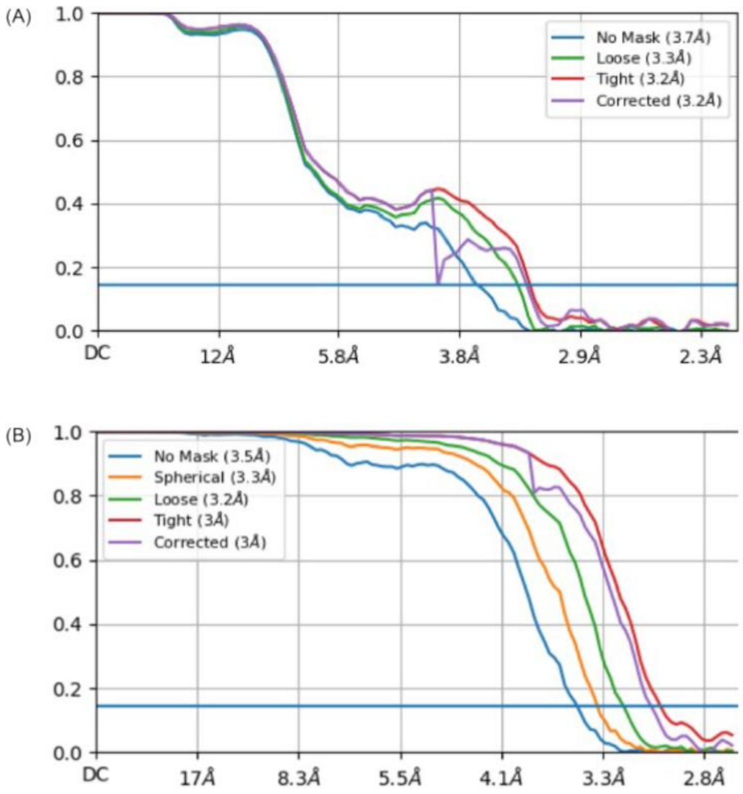

Supplemental Figure S4. FSC plots of the finalized maps (A) DOPG-SEn map and (B) DOPG-Sen-hPg map. The values shown contain the final fitted resolutions for both maps using the gold standard FSC value of 0.143.

Supplemental Figure S5

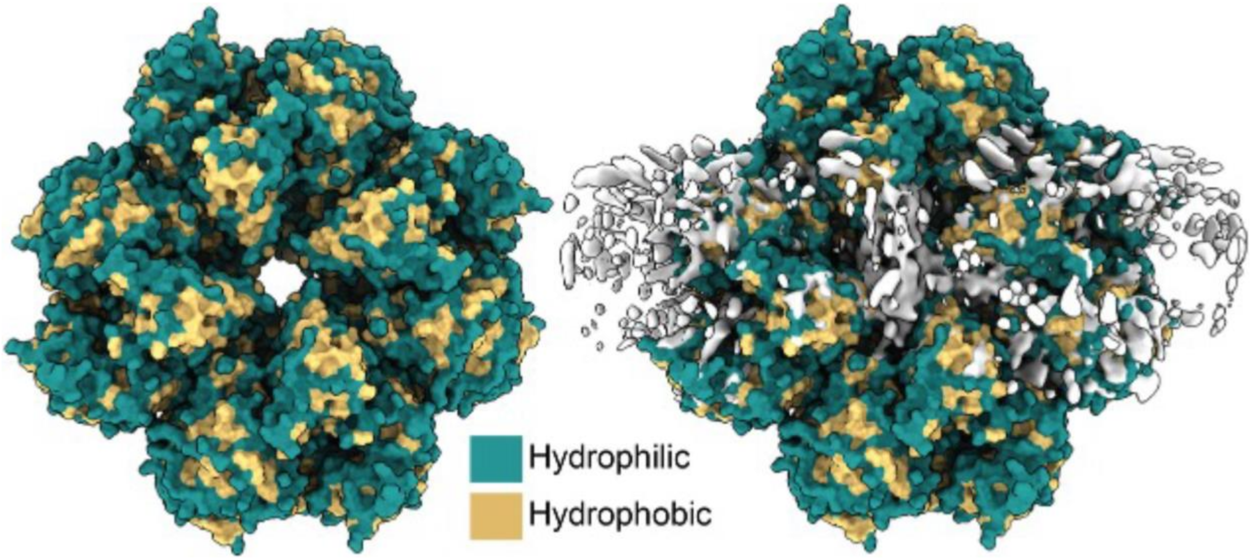

Supplemental Figure S5. SEn model with the surface charge displayed, green for hydrophilic residues and gold for hydrophobic residues. The surface was then superimposed on the lipid map colored in white to show the localization of the lipid.

Supplemental Figure S6

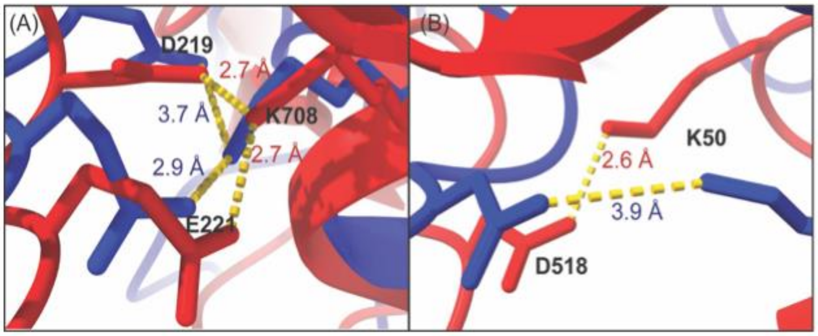

Supplemental Figure S6. The key residues that maintain the closed conformation of hPg. (A) H-bonding between D219, E221, and K708, as well as (B) interactions between K50 and D518. These interactions were obtained by overlaying the structure from X-ray PDB 4DUR (red) and PDB 8UQ6 (blue).
